# Sexual dimorphism in the long-term stability (10 years) of skeletal Class III treatment

**DOI:** 10.1186/s40510-021-00360-w

**Published:** 2021-06-21

**Authors:** Natalia Tejedor, Conchita Martín, José Antonio Alarcón, María Dolores Oteo-Calatayud, Juan Carlos Palma-Fernández

**Affiliations:** 1 Department of Clinical Dental Specialties, Section of Orthodontics, Faculty of Dentistry, Complutense University, Plaza de Ramón y Cajal s/n, 28040 Madrid, Spain; 2 BIOCRAN Research Group, Faculty of Dentistry, Complutense University of Madrid, Madrid, Spain; 3 Department of Stomatology, Section of Orthodontics, Faculty of Odontology, University of Granada, Campus Universitario de Cartuja, s/n, 18071 Granada, Spain

**Keywords:** Skeletal Class III, Treatment, Long-term, Stability, Sexual dimorphism

## Abstract

**Background:**

Class III malocclusion is associated with high sexual dimorphism, especially in individuals older than 13 years of age, with significant differences in growth between males and females during the pubertal and postpubertal stages, and in adulthood. The aim of this research was to examine differences between males and females in long-term stability (10 years) of treatment for skeletal Class III malocclusion.

**Methods:**

Thirty patients (15 males and 15 females) with skeletal Class III malocclusion, who had been treated with rapid maxillary expansion (RME) combined with face mask protraction followed by fixed appliances, were selected sequentially. Thirty patients (15 males and 15 females) with skeletal Class I and mesofacial patterns treated only with fixed appliances for dental problems served as the control group. Differences between groups and sexes were evaluated using lateral cephalograms taken at the start of treatment (T0), immediately after the end of treatment (T1), and after 10 years (T2). The long-term treatment success rate was calculated.

**Results:**

Ten years after Class III treatment, overjet and overbite relapse occurred similarly in females (− 0.68 ± 0.7 mm; − 0.38 ± 0.75 mm, respectively) and males (− 1.09 ± 1.47 mm; − 0.64 ± 0.9 mm, respectively); the ANB angle and Wits appraisal became significantly more negative in males (− 1.37 ± 1.06°; − 2.7 ± 2.53 mm) than in females (− 0.18 ± 1.26°; − 0.46 ± 1.94 mm). The success rate was 73.3% in males and 80% in females.

**Conclusions:**

Significant differences in the long-term stability of Class III treatment outcomes have been found between males and females, with a larger skeletal Class III relapse and lower long-term success rates in males.

## Background

There is a wide variety of individual responses to skeletal Class III malocclusion treatment, making it difficult to predict the outcome. In some cases, the outcome is unsatisfactory, despite an adequate orthodontic treatment. The literature indicates the advantages of early treatment [1–3] since the outcomes are more favourable, such as achieving larger skeletal effects, less dentoalveolar effects, and less mandibular posterior rotation [4–9]. Among the benefits achieved by an early intervention, one should consider the decrease in the negative effect on self-esteem of children and adolescents, triggered by a Class III malocclusion which has an aesthetic effect, therefore the future need of orthognatic surgery is reduced [10, 11].

Short-term stability of Class III malocclusion treatment has been widely studied; however, very few studies have focused on the long-term stability of outcomes [12]. Most studies describe the outcomes obtained once the orthopaedic treatment has ended [6, 13–16] or after a second phase with fixed appliances [4, 5, 17, 18]. A small number of studies describe medium-term stability (2–5 years) [19–23]. However, studies on short- and medium-term stability of Class III treatment outcomes are insufficient; many studies indicate a high mandibular growth potential in Class III malocclusion, higher than in subjects with normal occlusion, and this late and unpredictable mandibular growth has been observed to cause Class III relapse [5, 18, 21, 22, 24].

Class III malocclusion is associated with high sexual dimorphism, especially in individuals older than 13 years of age, with significant differences in growth between males and females during the pubertal and postpubertal stages, and in adulthood [20], which should be considered in research on class III malocclusion.

After reviewing the literature, there is an obvious need for longer follow-up studies with skeletal Class III patients to evaluate the stability of treatment outcomes and the effect of sex in the long-term stability [25].

Therefore, the present study aims to cephalometrically assess the differences between males and females in long-term stability (10 years) of skeletal Class III treatment outcomes with rapid maxillary expansion (RME) combined with face mask protraction and followed by fixed appliances.

## Methods

### Samples

In this retrospective study, the post-treatment archive from the Department of Orthodontics of the School of Dentistry, Complutense University, Madrid, Spain, was searched to identify all subjects meeting the following inclusion criteria at the beginning of treatment (T0): skeletal III malocclusion (ANB angle ≤ 0° and Wits appraisal ≤ − 2.0 mm), class III molar relationship, anterior crossbite or edge-to-edge incisal relationship, prepubertal skeletal maturation (cervical stage [CS] CS1–CS3 )[26] and European white descent. At the end of treatment (T1), postpubertal skeletal maturation (CS5–CS6) was a requirement too [26]. Exclusion criteria included congenitally missing, supernumerary, or extracted teeth; craniofacial disorders; temporomandibular joint dysfunction; patients classified as surgical cases; and previous treatment. The entrance criteria for the control group were: skeletal Class I (ANB angle between 0° and 3°, and Wits appraisal between -2.0 mm and 2.0 mm), mesofacial growth pattern (mandibular plane angle [MPA] between 20° and 28°), European white descent and with a non-extraction treatment with fixed appliances for moderate dental problems, including molar class I and minor crowding.

After reviewing more than 800 records of patients treated between 1995 and 2005, a total of 120 class III and 160 class I patients were identified. Once the availability of adequate records and the class III treatment protocol rendered was checked, a sample of 30 patients (15 males and 15 females) with skeletal Class III malocclusion, who had been treated with a combination of RME and protraction facemask followed by a second phase with fixed appliances were selected. As control group, 30 patients (15 males and 15 females) that were matched to the Class III group according to age, origin, skeletal maturity at all observation periods, duration of observation intervals were selected.

The sample size was calculated for a statistical power of 80%, an alpha error value of 0.05, and 95% confidence interval, resulting in a minimum sample size of 18 subjects per group.

The study protocol was approved by the Clinical Research Ethics Committee of the San Carlos Clinical Hospital (Madrid, reference number “C.P. - C.I. 12/152-E”. All subjects were informed of the characteristics of the study and agreed to participate by signing an informed consent form.

### Class III malocclusion treatment protocol

Subjects with Class III malocclusion were treated in two phases. The 1st phase comprised RME and protraction facemask. Treatment was started by cementing a Hyrax expander (Dentaurum, Ispringen, Germany) with anterior protraction hooks soldered at the level of the canines. Patients’ parents were instructed to activate the screw with a quarter-turn (0.25 mm) twice a day until an overexpansion of 3 mm was achieved.

Immediately after expansion, the protraction facemask was placed on the patient by adjusting the elastics from the expander hooks to the mask hooks resulting in a downward and forward direction of the traction vector, at an angle of approximately 30° relative to the occlusal plane. The elastics were used with a traction force of 400 g per side and patients were instructed to wear the mask approximately 13–14 h a day. This first phase of orthopaedic treatment, which lasted 1.5 to 2 years on average, aimed to achieve a positive overjet of at least 3–4 mm and a class II molar relationship of 1–2 mm. Upon achieving these goals, the first phase was completed. Recall visits were scheduled to evaluate the stability of the first phase results until the tooth replacement was over.

Orthodontic treatment continued in a second phase by using fixed appliances (straight arch system with 0.018 slot brackets, Hilgers' prescription; Ormco, Glendora, Calif) for 2 to 2.5 years. After this treatment, in the retention phase, a removable Hawley plate was used in the upper arch full time for 6 months; and right after these months passed, it was used during night time. A canine-to-canine lingual retainer was used in the lower arch. The average treatment period in the Class III group was 5.58 ± 2 years.

### Class I control group treatment protocol

Orthodontic treatment consisted of the use of upper and lower fixed appliances, using the same prescription as in the experimental group, slight lower anterior stripping and settling elastics. The retention protocol was the same as in the Class III malocclusion group described above.

### Measurement method

Both groups were evaluated by lateral cephalograms at the beginning of treatment (T0), immediately after the end of treatment (T1), and after a period of 10 years (T2). All patients reached the end of treatment (T1) in the postpubertal period (CS5–CS6) [26] and had completed active circumpubertal craniofacial growth (CS6) [26] long before T2. Lateral cephalograms were scanned with a millimetre ruler using the Epson Scan software for correct calibration. Digital images were traced using the Dolphin Imaging Version 11.5 Software, Chatsworth, Calif. Cephalometric analysis performed for the three study time points (T0/T1/T2) included cephalometric measurements of Steiner, Ricketts, McNamara, Jarabak, and Wits appraisal, generating 29 variables.

### Statistical analysis

Descriptive statistics were calculated for the different observation time points in T0, T1, T2, and the intervals T0–T1 and T1–T2, separating both the experimental and control groups by sex. After establishing the normal distribution of variables by means of the Kolmogorov-Smirnov test, repeated-measures ANOVA was used to compare each cephalometric variable analysed in the three observation periods, separating each group by sex. Thus, the evolution of each cephalometric variable over time was analysed in females and males with Class III and in those with Class I. Statistical comparison between females/males with Class III and those in the control group for time points T0, T1, T2, and intervals T0–T1 and T1–T2 was performed using Student’s *t* test for independent samples. The level of statistical significance was established at 95% (*p* ≤ 0.05).

The treatment success rate was calculated in the long term (T2). As consistently reported in the literature, an unsatisfactory outcome in the treatment of Class III malocclusion is defined by a class III molar relationship and a negative overjet in any incisor [1, 21, 22, 27, 28].

The method error was determined by cephalometric tracing on lateral cephalograms of 20 randomly selected patients (10 patients with Class III and 10 patients in the control group) at the three study time points (T0, T1, T2); therefore, 60 lateral cephalograms were traced again 2 weeks later by the same examiner. Intraclass correlation coefficient ranged from 0.839 for overbite at T1 to 0.998 for overjet at T0, showing high intraclass correlation.

## Results

Table 1 shows the values of all cephalometric variables analysed in males of the two groups (Class III and Class I), at the three study time points (T0, T1, T2). At the beginning of treatment (T0), males with Class III exhibit a smaller ANB angle (0.72 ± 1.66°) and Wits appraisal (− 6.31 ± 2.06 mm), increased maxillomandibular difference (29.17 ± 4.55 mm), less overjet (0.23 ± 2.35 mm) and overbite (1.49 ± 2.12 mm), a more negative molar relationship (more class III molar relationship) (-3.26 ± 1.82 mm), and a more protruded lower incisor (3.10 ± 2.38 mm) but more lingually inclined (87.28 ± 4.64°) than in males with Class I. At the end of the second treatment phase (T1), comparisons between males also showed significant differences. Males with Class III exhibit a more negative Wits appraisal (− 3.81 ± 2.82 mm), increased maxillomandibular difference (34.45 ± 3.76 mm), less overbite (1.54 ± 0.53 mm), larger interincisal angle (129.52 ± 5.98°) and an lower incisor in linguoversion (incisor to mandibular plane angle [IMPA] 86.90 ± 5.81°) compared to males with Class I. Ten years after the end of treatment (T2), males with Class III exhibit a more prominent Pogonion (Pg) (3.58 ± 7.71 mm), a more negative ANB angle (− 0.16 ± 1.92°) and Wits appraisal (− 6.52 ± 1.91 mm), increased maxillomandibular difference (40.09 ± 6.12 mm), less overjet (1.26 ± 1.63 mm) and less overbite (0.90 ± 0.90 mm), and a lower incisor more lingually inclined (IMPA: 85.76 ± 4.39°) than in males with Class I.

**Table 1:** Descriptive statistics and comparasions between male experimental (EG) and control (CG) groups, before treatment (T0), at the end of second treatment phase (T1) and 10 years after treatment (T2). *Po* Porion, *Co* Condyleon, *perp* perpendicular, *Co-Gn* Condyleon-Gnation, *Pg* Pogonion, *FH* Frankfurt Horizontal, *SN* Sella-Nasion, *MPA* mandibular plane angle, *Me* Menton, *S* Sella, *Go* Gonion, *ANS* anterior nasal spine, *U1* upper incisor, *L1* lower incisor, *E plane* Esthetic plane. * *p* < 0.05; ** *p* < 0.01; *** *p* < 0.001

Cephalometric measures	T0				T1				T2				T0	T1	T2
	EG		CG		EG		CG		EG		CG		EG vs. CG		
	Mean	SD	Mean	SD	Mean	SD	Mean	SD	Mean	SD	Mean	SD	(***t*** test)		
**Cranial base**															
Cranial flexure (°)	29.38	3.50	28.41	2.14	28.92	2.70	27.84	2.15	28.89	1.84	28.53	1.97	NS	NS	NS
Po localization (mm)	− 43.20	4.15	− 43.81	2.56	− 45.31	4.43	− 44.76	2.24	− 47.99	4.93	− 45.73	3.25	NS	NS	NS
Anterior cranial base (mm)	70.92	4.20	72.57	2.88	74.21	5.71	74.13	2.83	76.92	6.53	78.00	1.87	NS	NS	NS
Posterior cranial base (mm)	31.97	2.61	33.46	2.75	34.67	2.67	36.03	2.74	38.18	5.10	37.73	2.84	NS	NS	NS
**Maxillary skeletal**															
Co-point A (mm)	86.64	5.84	90.00	2.63	91.74	6.34	92.36	2.81	97.58	11.02	96.88	2.08	NS	NS	NS
SNA (°)	79.55	3.88	80.84	2.14	80.04	3.54	80.78	2.54	80.10	3.55	80.35	2.92	NS	NS	NS
Point A-Nasion perp (mm)	− 0.47	2.71	0.00	2.73	− 0.12	2.75	− 1.55	2.85	0.46	4.34	− 1.93	2.69	NS	NS	NS
Maxillary depth (°)	89.52	2.14	89.94	2.57	89.75	3.40	88.60	2.40	90.46	3.78	88.87	2.64	NS	NS	NS
**Mandibular skeletal**															
Co-Gn (mm)	115.80	6.23	114.37	3.43	126.39	8.22	122.01	4.93	135.33	9.16	130.74	4.52	NS	NS	NS
SNB (°)	78.84	3.86	78.34	2.16	78.90	3.69	79.09	3.33	80.55	4.17	78.56	3.50	NS	NS	NS
Pg-Nasion perp (mm)	− 0.48	6.85	− 2.96	4.34	− 0.26	6.30	− 3.36	4.80	3.58	7.71	− 2.20	5.24	NS	NS	*
**Maxillary/mandibular**															
Wits appraisal (mm)	− 6.31	2.06	− 0.40	1.08	− 3.81	2.82	− 0.66	1.26	− 6.52	1.91	0.07	1.75	***	**	***
ANB (°)	0.72	1.66	2.44	0.65	1.20	1.42	1.53	1.28	− 0.16	1.92	1.74	0.74	**	NS	**
Maxillary/mandibibular difference (mm)	29.17	4.55	25.04	2.84	34.45	3.76	29.41	2.81	40.09	6.12	33.83	4.31	**	***	**
**Vertical skeletal**															
FH-occlusal plane (°)	9.17	3.59	8.16	2.90	7.38	3.75	7.54	2.89	6.29	4.13	6.31	3.22	NS	NS	NS
SN-palatal plane (°)	9.44	4.49	7.31	2.51	8.64	3.87	6.34	3.37	8.08	4.79	8.64	2.88	NS	NS	NS
MPA (°)	27.38	4.33	26.12	2.47	27.00	4.92	26.60	3.21	25.03	5.96	24.78	3.69	NS	NS	NS
Nasion-Me (mm)	118.34	5.78	118.00	6.47	128.96	8.29	125.75	5.93	139.20	18.63	134.21	5.09	NS	NS	NS
S-Go (mm)	71.76	6.66	72.34	6.19	79.86	5.85	78.52	6.47	84.95	8.51	87.13	5.06	NS	NS	NS
ANS-Me (mm)	67.31	4.41	67.03	3.47	73.02	5.29	70.03	3.95	78.84	10.84	73.15	3.33	NS	NS	NS
Gonial angle (°)	130.54	6.75	124.58	4.49	125.86	8.01	122.64	4.64	124.87	6.12	121.44	5.48	NS	NS	NS
**Facial pattern**															
S-Go/Nasion-Me (%)	59.95	4.60	61.14	3.82	61.90	4.60	62.84	4.33	63.57	5.08	63.82	4.18	NS	NS	NS
Facial axis (°)	88.78	3.61	89.98	2.44	87.90	4.96	89.72	3.29	89.78	4.55	90.52	3.52	NS	NS	NS
**Interdental**															
Overjet (mm)	0.23	2.35	4.16	1.12	2.36	0.39	2.10	1.10	1.26	1.63	3.73	0.85	***	NS	***
Overbite (mm)	1.49	2.12	3.08	1.40	1.54	0.53	1.93	0.48	0.90	0.90	2.78	1.04	*	*	***
Interincisal angle (°)	134.90	8.67	133.32	10.21	129.52	5.98	124.60	5.77	133.07	6.23	130.94	7.05	NS	*	NS
Molar relationship (mm)	− 3.26	1.82	− 0.59	1.00	− 2.54	1.09	− 2.44	0.66	− 3.30	1.65	− 2.57	0.72	***	NS	NS
**Dentoalveolar**															
U1 to point A to Nasion perp (mm)	4.54	1.53	4.62	1.57	6.22	1.29	5.89	1.24	7.36	1.26	7.02	1.94	NS	NS	NS
U1 to FH (°)	111.99	5.63	111.76	6.06	116.81	3.56	115.63	3.65	116.75	3.45	115.65	3.45	NS	NS	NS
L1 to A-Pg (mm)	3.10	2.38	1.22	1.89	3.62	1.65	3.55	1.07	3.95	1.64	2.83	2.06	*	NS	NS
L1 to MPA (°)	87.28	4.64	92.58	3.30	86.90	5.81	93.98	2.66	85.76	4.39	93.47	2.79	**	***	***
**Soft tissue relationship**															
Lower lip to E plane (mm)	− 0.82	2.07	− 0.69	1.98	− 1.75	2.51	− 1.61	1.77	− 3.19	2.86	− 3.37	1.62	NS	NS	NS
Upper lip to E plane (mm)	− 2.77	2.79	− 2.14	2.13	− 3.74	3.11	− 4.06	1.51	− 6.38	3.16	− 6.05	1.38	NS	NS	NS
Nasiolabial angle (°)	98.70	7.41	96.26	2.96	98.26	6.10	97.90	3.39	99.33	7.00	97.46	4.23	NS	NS	NS

Table 2 shows the same outcomes in females. At T0, compared to females with Class I, females with Class III exhibit shorter anterior cranial base (ACB) (66.04 ± 3.69 mm), reduced maxillary length (Co-A) (77.89 ± 4.27 mm), an increased SNB angle (79.98 ± 2.46°), more negative ANB angle (-0.21 ± 2.10°) and Wits appraisal (-7.51 ± 2.70 mm), an augmented occlusal plane inclination (10.34 ± 2.97°), a larger gonial angle (130.54 ± 6.75°), lower posterior facial height (PFH) (65.72 ± 3.45 mm), less overjet (− 1.31 ± 2.30 mm), less overbite (0.28 ± 1.52 mm), more negative molar relationship (more class III molar relationship) (− 4.26 ± 2.16 mm), a more retruded upper incisor (3.50 ± 2.04 mm), more protruded lower incisor (3.01 ± 1.74 mm) but in linguoversion (IMPA: 82.54 ± 6.64°), and more retruded upper lip (− 4.08 ± 2.26 mm). At T1, females with Class III exhibit an increased mandibular length (Co-Gn) (118.11 ± 4.83 mm), a larger SNB angle (79.99 ± 3.01°), more negative Wits appraisal (− 4.34 ± 2.20 mm), and lower incisor in linguoversion (IMPA: 83.86 ± 6.44°), compared to females with Class I. At T2, females with Class III continue to show a larger mandibular length (Co-Gn) (124.60 ± 6.33 mm), a larger SNB angle (80.40 ± 3.80°), more negative Wits appraisal (− 4.80 ± 2.89 mm), less overjet (2.08 ± 1.02 mm), less overbite (1.10 ± 1.11 mm), and lower incisor more lingually inclined (85.13 ± 7.31°) than in females with Class I.

**Table 2:** Descriptive statistics and comparasions between female experimental (EG) and control (CG) groups, before treatment (T0), at the end of second treatment phase (T1) and 10 years after treatment (T2). *Po* Porion, *Co* Condyleon, *perp* perpendicular, *Co-Gn* Condyleon-Gnation, *Pg* Pogonion, *FH* Frankfurt Horizontal, *SN* Sella-Nasion, *MPA* mandibular plane angle, *Me* Menton, *S* Sella, *Go* Gonion, *ANS* anterior nasal spine, *U1* upper incisor, *L1* lower incisor, *E plane* Esthetic plane. * *p* < 0.05; ** *p* < 0.01; *** *p* < 0.001

Cephalometric measures	T0				T1				T2				T0	T1	T2
	EG		CG		EG		CG		EG		CG		EG vs. CG		
	Mean	SD	Mean	SD	Mean	SD	Mean	SD	Mean	SD	Mean	SD	(***t*** test)		
**Cranial base**															
Cranial flexure (°)	28.01	2.36	28.20	1.82	28.63	2.23	28.93	1.95	28.99	2.00	29.50	1.69	NS	NS	NS
Po localization (mm)	− 40.80	2.40	− 41.18	2.47	− 41.60	3.11	− 42.05	2.39	− 41.04	3.59	− 42.16	2.47	NS	NS	NS
Anterior cranial base (mm)	66.04	3.69	68.40	2.41	69.97	3.48	70.57	2.58	72.06	3.81	72.12	2.79	*	NS	NS
Posterior cranial base (mm)	29.60	2.27	31.60	2.43	33.34	2.94	33.10	2.28	33.54	3.41	33.76	2.14	*	NS	NS
**Maxillary skeletal**															
Co-point A (mm)	77.89	4.27	80.82	3.38	84.83	5.06	83.92	3.77	88.10	3.63	87.09	4.72	*	NS	NS
SNA (°)	79.87	2.77	78.60	3.40	80.28	2.82	77.85	3.90	80.74	3.52	78.18	4.07	NS	NS	NS
Point A-Nasion perp (mm)	− 0.23	3.33	− 0.41	2.31	0.35	3.32	− 0.64	3.97	1.21	3.71	0.61	3.06	NS	NS	NS
Maxillary depth (°)	89.77	3.77	89.57	2.33	90.37	3.40	89.35	3.86	91.20	3.56	90.59	2.97	NS	NS	NS
**Mandibular skeletal**															
Co-Gn (mm)	106.18	5.41	108.06	7.07	118.11	4.83	114.44	4.81	124.60	6.33	119.88	5.69	NS	*	*
SNB (°)	79.98	2.46	76.53	3.31	79.99	3.01	76.64	3.67	80.40	3.80	76.80	4.33	**	*	*
Pg-Nasion perp (mm)	0.66	4.51	− 2.90	5.22	1.92	6.35	− 1.40	6.71	4.57	6.66	1.96	6.28	NS	NS	NS
**Maxillary/mandibular**															
Wits appraisal (mm)	− 7.51	2.70	0.18	2.00	− 4.34	2.20	− 0.56	1.59	− 4.80	2.89	− 0.40	1.98	***	***	***
ANB (°)	− 0.21	2.10	2.06	1.06	0.51	2.21	1.20	1.44	0.33	3.05	1.44	1.36	**	NS	NS
Maxillary/mandibular difference (mm)	28.26	3.81	26.88	5.06	33.28	4.08	30.50	3.65	36.50	6.08	32.77	3.45	NS	NS	NS
**Vertical skeletal**															
FH-occlusal plane (°)	10.34	2.97	6.97	3.13	6.19	3.37	5.35	3.76	4.97	4.20	2.97	3.53	**	NS	NS
SN-palatal plane (°)	8.32	2.36	7.38	2.67	7.86	3.00	8.87	3.75	8.31	2.85	8.06	2.30	NS	NS	NS
MPA (°)	27.12	4.53	25.16	4.23	26.33	5.83	24.60	4.88	24.86	5.49	22.66	5.53	NS	NS	NS
Nasion-Me (mm)	107.94	7.17	112.14	8.00	120.90	5.84	118.66	5.33	125.14	7.02	122.23	5.91	NS	NS	NS
S-Go (mm)	65.72	3.45	70.26	6.37	75.45	4.27	74.82	5.05	79.37	6.40	78.42	4.58	*	NS	NS
ANS-Me (mm)	61.07	4.63	64.57	5.24	68.43	5.36	67.94	4.81	71.60	6.59	69.84	5.31	NS	NS	NS
Gonial angle (°)	130.54	6.75	124.58	4.49	125.86	8.01	122.64	4.64	124.87	6.12	121.44	5.48	**	NS	NS
**Facial pattern**															
S-Go/Nasion-Me (%)	61.10	4.55	62.68	4.18	62.58	5.30	63.55	4.58	64.04	4.69	64.32	5.09	NS	NS	NS
Facial axis (°)	91.66	3.90	89.74	3.76	90.33	5.42	89.28	4.12	91.21	5.48	90.34	4.61	NS	NS	NS
**Interdental**															
Overjet (mm)	− 1.31	2.30	4.04	0.94	2.76	0.71	2.90	0.93	2.08	1.02	3.10	0.70	***	NS	**
Overbite (mm)	0.28	1.52	2.34	2.06	1.48	0.74	1.48	0.78	1.10	1.11	2.23	1.12	**	NS	*
Interincisal angle (°)	141.39	14.33	133.86	9.44	130.78	6.19	126.96	8.25	131.44	8.56	127.77	9.37	NS	NS	NS
Molar relationship (mm)	− 4.26	2.16	− 0.67	1.21	− 2.01	1.12	− 1.74	0.74	− 2.56	1.32	− 1.76	0.82	***	NS	NS
**Dentoalveolar**															
U1 to point A to Nasion perp (mm)	3.50	2.04	5.40	2.05	6.60	1.58	6.02	2.35	7.48	2.30	7.16	2.03	*	NS	NS
U1 to FH (°)	102.26	26.49	113.80	6.51	119.05	5.43	117.46	8.47	118.56	6.79	118.27	8.88	NS	NS	NS
L1 to A-Pg	3.01	1.74	1.29	2.53	3.02	1.98	2.94	1.84	3.55	2.62	3.07	2.21	*	NS	NS
L1 to MPA (°)	82.54	6.64	87.85	5.43	83.86	6.44	91.01	6.04	85.13	7.31	92.16	4.23	*	**	**
**Soft tissue realtionship**															
Lower lip to E plane (mm)	− 0.64	2.04	− 0.40	1.76	− 1.50	2.49	− 1.24	2.29	− 2.44	3.21	− 2.70	2.78	NS	NS	NS
Upper lip to E plane (mm)	− 4.08	2.26	− 2.42	1.92	− 4.26	2.32	− 3.86	1.96	− 5.83	2.94	− 5.72	1.81	*	NS	NS
Nasiolabial angle (°)	102.43	6.25	103.13	6.45	99.40	4.99	102.43	9.02	100.16	6.49	102.26	6.71	NS	NS	NS

Table 3 shows the differences observed during the periods T0–T1 and T1–T2 between females and males in the Class I group. During the treatment period (T0–T1), cranial deflection increases in females (0.73 ± 1.70°) and decreases in males (− 0.56 ± 1.49°), the distance from Pg to the vertical plane increases more in females (1.50 ± 3 mm), and the palatal plane angle (PP-SN) increases in females (1.49 ± 3.15°) and decreases in males (− 0.96 ± 1.84°). In the T1-T2 interval, ACB increases more in males (3.87 ± 2.64 mm), the distance from point A to the vertical plane increases in females (1.26 ± 1.55 mm) and decreases in males (− 0.38 ± 1.19 mm), mandibular length increases more in males (8.72 ± 3.61 mm) along with maxillomandibular difference (4.42 ± 3.20 mm), the palatal plane angle (PP-SN) is reduced in females (− 0.80 ± 2.67°) and increased in males (2.29 ± 2.94°), the anterior and posterior facial heights (AFH and PFH) increase more in males (AFH: (38.46 ± 3.08 mm) and PFH: (8.61 ±3.56 mm)), overjet remains the same in females and increases more in males(1.62 ± 1.34 mm); finally, the interincisal angle increases more in males (6.34 ± 4.41°).

**Table 3:** Comparison between females and males during the periods T0–T1 and T1–T2 in the Class I group. *Po* Porion, *Co* Condyleon, *perp* perpendicular, *Co-Gn* Condyleon-Gnation, *Pg* Pogonion, *FH* Frankfurt Horizontal, *SN* Sella-Nasion, *MPA* mandibular plane angle, *Me* Menton, *S* Sella; *Go* Gonion, *ANS* anterior nasal spine, *U1* upper incisor, *L1* lower incisor, *E plane* Esthetic plane. * *p* < 0.05; ** *p* < 0.01; *** *p* < 0.001

Cephalometric measures	T0–T1				T1–T2				Female vs. male T0–T1	Female vs. male T1–T2
	Female		Male		Female		Male		***t*** test	t-test
	Mean	SD	Mean	SD	Mean	SD	Mean	SD	Significance	Significance
**Cranial base**										
Cranial flexure (°)	0.73	1.70	− 0.56	1.49	0.56	1.38	0.68	1.46	*	NS
Po localization (mm)	− 0.87	2.30	− 0.95	1.51	− 0.10	1.71	− 0.96	1.95	NS	NS
Anterior cranial base (mm)	2.16	2.55	1.56	2.05	1.60	1.41	3.87	2.64	NS	**
Posterior cranial base (mm)	1.50	2.31	2.56	1.69	0.66	1.42	1.70	1.53	NS	NS
**Maxillary skeletal**										
Co-point A (mm)	3.09	2.16	2.36	2.24	3.17	2.16	4.51	2.08	NS	NS
SNA (°)	− 0.75	2.07	− 0.06	1.04	0.32	1.22	− 0.42	1.08	NS	NS
Point A-Nasion perp (mm)	− 0.23	2.23	− 1.56	1.10	1.26	1.55	− 0.38	1.19	NS	**
Maxillary depth (°)	− 0.22	2.09	− 1.34	0.84	1.24	1.63	0.26	1.26	NS	NS
**Mandibular skeletal**										
Co-Gn (mm)	6.38	4.06	7.64	3.77	5.44	3.15	8.72	3.61	NS	*
SNB (°)	0.11	1.60	0.74	1.59	0.16	1.47	− 0.53	1.71	NS	NS
Pg-Nasion perp (mm)	1.50	3.00	− 0.40	1.83	3.37	4.28	1.16	2.30	*	NS
**Maxillary/mandibular**										
Wits appraisal (mm)	− 0.74	1.21	− 0.26	0.82	0.16	0.80	0.73	1.25	NS	NS
ANB (°)	− 0.85	1.18	− 0.91	1.12	0.23	0.88	0.20	1.16	NS	NS
Maxillary/mandibibular difference (mm)	3.62	2.20	4.37	2.16	2.26	1.88	4.42	3.20	NS	*
**Vertical skeletal**										
FH-occlusal plane (°)	− 1.62	2.64	− 0.62	2.78	− 2.38	2.64	− 1.22	2.26	NS	NS
SN-palatal plane (°)	1.49	3.15	− 0.96	1.84	− 0.80	2.67	2.29	2.94	*	**
MPA (°)	− 0.56	2.22	0.47	1.99	− 1.93	2.81	− 1.82	2.18	NS	NS
Nasion-Me (mm)	6.52	5.54	7.75	4.87	3.56	2.52	8.46	3.08	NS	***
S-Go (mm)	4.55	4.64	6.17	3.36	3.60	3.36	8.61	3.56	NS	***
ANS-Me (mm)	3.37	3.25	3.00	2.97	1.90	1.86	3.12	2.28	NS	NS
Gonial angle (°)	− 1.94	1.99	− 1.74	2.69	− 1.20	4.74	− 3.90	6.82	NS	NS
**Facial pattern**										
S-Go/Nasion-Me (%)	0.86	2.30	1.70	1.56	0.77	2.03	0.97	2.46	NS	NS
Facial axis (°)	− 0.46	1.56	− 0.26	1.53	1.06	1.49	0.79	1.20	NS	NS
**Interdental**										
Overjet (mm)	− 1.14	1.48	− 2.06	1.48	0.20	1.15	1.62	1.34	NS	**
Overbite (mm)	− 0.85	1.89	− 1.14	1.50	0.74	1.31	0.84	1.02	NS	NS
Interincisal angle (°)	− 6.90	11.73	− 8.72	13.10	0.81	8.79	6.34	4.41	NS	*
Molar relationship (mm)	− 1.06	1.23	− 1.85	1.27	− 0.02	0.81	− 0.12	1.11	NS	NS
**Dentoalveolar**										
U1 to point A to Nasion perp (mm)	0.62	2.71	1.27	1.35	1.14	0.85	1.12	1.19	NS	NS
U1 to FH (°)	3.66	10.42	3.86	7.09	0.80	5.76	0.02	2.43	NS	NS
L1 to A-Pg (mm)	1.65	2.07	2.33	1.55	0.12	1.47	− 0.72	1.52	NS	NS
L1 to MPA (°)	3.16	6.18	1.40	3.57	1.15	4.41	− 0.51	1.81	NS	NS
**Soft tissue relationship**										
Lower lip to E plane (mm)	− 0.83	2.06	− 0.92	1.42	− 1.46	2.30	− 1.76	0.85	NS	NS
Upper lip to E plane (mm)	− 1.44	2.12	− 1.92	1.37	− 1.86	2.03	− 1.98	0.95	NS	NS
Nasiolabial angle (°)	− 0.70	6.63	1.63	2.04	− 0.16	5.68	− 0.43	2.10	NS	NS

Table 4 shows the comparison of the differences between males and females in the Class III group during the periods T0–T1 and T1–T2. In the T0–T1 period, the only variable showing a significant difference is overjet. Women show larger changes in overjet, with better outcomes during treatment than males (women: 4.07 ± 2.44 mm/men: 2.12 ± 2.27 mm). In the T1–T2 period, the position of the Porion (Po) increases slightly in females (0.56 ± 3.25 mm) and decreases in males (− 2.68 ± 5.12 mm), the posterior cranial base (PCB) grows more in males (females: 0.20 ± 2.63 mm/males: 3.51 ± 5.29 mm), and the Wits appraisal and ANB variables become more negative in males (Wits: − 2.70 ± 2.53 mm/ANB: − 1.37 ± 1.06°) than in females (Wits: − 0.46 ± 1.94 mm/ANB: − 0.18 ± 1.27°).

**Table 4:** Comparison between females and males during the periods T0–T1 and T1–T2 in the Class III group. *Po* Porion, *Co* Condyleon, *perp* perpendicular, *Co-Gn* Condyleon-Gnation, *Pg* Pogonion, *FH* Frankfurt Horizontal, *SN* Sella-Nasion, *MPA* mandibular plane angle, *Me* Menton, *S* Sella, *Go* Gonion, *ANS* anterior nasal spine, *U1* upper incisor, *L1* lower incisor, *E plane* Esthetic plane. * *p* < 0.05

Cephalometric measures	T0–T1				T1–T2				Female vs. male T0–T1	Female vs. male T1–T2
	Female		Male		Female		Male		***t*** test	***t*** test
	Mean	SD	Mean	SD	Mean	SD	Mean	SD	Significance	Significance
**Cranial base**										
Cranial flexure (°)	0.62	2.67	− 0.46	1.26	0.36	2.14	− 0.02	1.55	NS	NS
Po localization (mm)	− 0.80	2.29	− 2.11	2.51	0.56	3.25	− 2.68	5.12	NS	*
Anterior cranial base (mm)	3.93	2.49	3.28	2.16	2.08	2.74	2.71	2.69	NS	NS
Posterior cranial base (mm)	3.74	1.90	2.70	1.92	0.20	2.63	3.51	5.29	NS	*
**Maxillary skeletal**										
Co-point A (mm)	6.94	3.97	5.10	3.66	3.26	3.67	5.84	10.81	NS	NS
SNA (°)	0.41	2.11	0.49	1.35	0.45	3.54	0.06	1.32	NS	NS
Point A-Nasion perp (mm)	0.58	2.14	0.35	2.16	0.86	1.83	0.58	2.97	NS	NS
Maxillary depth (°)	0.60	2.57	0.23	2.20	0.82	1.64	0.70	2.64	NS	NS
**Mandibular skeletal**										
Co-Gn (mm)	11.92	5.13	10.58	6.77	6.49	5.98	8.94	9.02	NS	NS
SNB (°)	0.00	2.28	0.06	1.12	0.41	2.59	1.65	2.32	NS	NS
Pg-Nasion perp (mm)	1.26	4.61	0.22	5.46	2.65	3.49	3.84	4.96	NS	NS
**Maxillary/mandibular**										
Wits appraisal (mm)	3.16	1.77	2.50	2.12	− 0.46	1.94	− 2.70	2.53	NS	*
ANB (°)	0.72	1.58	0.48	1.84	− 0.18	1.27	− 1.37	1.06	NS	*
Maxillary/mandibibular difference (mm)	5.02	3.50	5.28	4.81	3.22	3.23	5.64	7.62	NS	NS
**Vertical skeletal**										
FH-occlusal plane (°)	− 4.14	3.10	− 1.78	4.22	− 1.22	2.46	− 1.09	3.55	NS	NS
SN-palatal plane (°)	− 0.46	2.19	− 0.80	2.05	0.45	2.12	− 0.55	3.05	NS	NS
MPA (°)	− 0.79	2.94	− 0.38	2.81	− 1.47	2.95	− 1.96	2.88	NS	NS
Nasion-Me (mm)	12.96	5.33	10.62	6.70	4.24	6.33	10.24	19.48	NS	NS
S-Go (mm)	9.72	3.50	8.10	5.16	3.92	3.59	5.09	5.45	NS	NS
ANS-Me (mm)	7.36	3.76	5.70	3.43	3.17	3.29	5.82	11.59	NS	NS
Gonial angle (°)	− 4.68	5.33	− 2.42	3.39	− 0.98	4.55	− 1.08	3.32	NS	NS
**Facial pattern**										
S-Go/Nasion-Me (%)	1.48	2.28	1.95	1.78	1.46	2.76	1.66	2.04	NS	NS
Facial axis (°)	− 1.33	2.86	− 0.88	2.27	0.88	4.30	1.87	2.68	NS	NS
**Interdental**										
Overjet (mm)	4.07	2.44	2.12	2.27	− 0.68	0.70	− 1.09	1.47	*	NS
Overbite (mm)	1.20	1.55	0.05	2.40	− 0.38	0.75	− 0.64	0.90	NS	NS
Interincisal angle (°)	− 10.61	14.34	− 5.38	6.06	0.66	6.45	3.55	5.92	NS	NS
Molar relationship (mm)	2.25	2.17	0.72	1.98	− 0.54	1.23	− 0.76	1.76	NS	NS
**Dentoalveolar**										
U1 to point A to Nasion perp (mm)	3.10	2.82	1.68	0.96	0.88	1,62	1.14	0.87	NS	NS
U1 to FH (°)	16.79	28.64	4.82	6.59	− 0.48	3.98	− 0.06	2.85	NS	NS
L1 to A-Pg (mm)	0.01	1.53	0.52	1.50	0.52	1.39	0.32	1.21	NS	NS
L1 to MPA (°)	1.32	5.75	− 0.38	2.86	1.27	3.72	− 1.13	4.20	NS	NS
**Soft tissue relationship**										
Lower lip to E plane (mm)	− 0.86	2.00	− 0.93	1.89	− 0.94	2.08	− 1.44	1.61	NS	NS
Upper lip to E plane (mm)	− 0.17	2.28	− 0.97	2.47	− 1.57	2.08	− 2.63	2.80	NS	NS
Nasiolabial angle (°)	− 3.03	4.36	− 0.43	5.09	0.76	4.74	1.06	4.36	NS	NS

Finally, Tables 5 and 6 show the comparison of the differences by sex between Class III and Class I malocclusion in periods T0–T1 and T1–T2, respectively. In the T0–T1 period (Table 5), in females with Class III malocclusion, there is a larger increase in PCB (3.74 ± 1.90 mm), maxillary length (6.94 ± 3.97 mm), mandibular length (11.92 ± 5.13 mm), and facial heights (anterior (12.96 ± 5.33 mm), posterior (9.72 ± 3.50 mm), and inferior (7.36 ± 3.76 mm)); Wits appraisal increases (3.16 ± 1.77 mm) while in Class I, it becomes slightly more negative (− 0.74 ± 1.21 mm); the ANB angle increases (0.72 ± 1.58°) while in Class I, it decreases (− 0.85 ± 1.18°); the oclusal plane angle (OP-FH) decreases more in Class III (− 4.14 ± 3.10°); overjet and overbite increase in Class III (overjet: 4.07 ± 2.44 mm/overbite: 1.20 ± 1.55 mm) and decrease in Class I (overjet: − 1.14 ± 1.48 mm/overbite: − 0.85 ± 1.89 mm); the molar relationship changes from class III to class I values in the Class III group, the upper incisor is more protruded (3.10 ± 2.82 mm) and the lower incisor less protruded (3.10 ± 2.82 mm) in Class III.

**Table 5:** Comparison of the differences by sex between Class III (EG, experimental group) and Class I (CG, control group) groups in period T0–T1. *Po* Porion, *Co* Condyleon, *perp* perpendicular, *Co-Gn* Condyleon-Gnation, *Pg* Pogonion, *FH* Frankfurt Horizontal, *SN* Sella-Nasion, *MPA* mandibular plane angle, *Me* Menton, *S* Sella, *Go* Gonion, *ANS* anterior nasal spine, *U1* upper incisor, *L1* lower incisor, *E plane* Esthetic plane. * *p* < 0.05; ** *p* < 0.01; *** *p* < 0.001

Cephalometric measures	EG				CG				T0–T1 (***t*** test EG vs. CG)	
	Females		Males		Females		Males			
	Mean	SD	Mean	SD	Mean	SD	Mean	SD	Significance	Significance
**Cranial base**										
Cranial flexure (°)	0.62	2.67	− 0.46	1.26	0.73	1.70	− 0.56	1.49	NS	NS
Po localization (mm)	− 0.80	2.29	− 2.11	2.51	− 0.87	2.30	− 0.95	1.51	NS	NS
Anterior cranial base (mm)	3.93	2.49	3.28	2.16	2.16	2.55	1.56	2.05	NS	*
Posterior cranial base (mm)	3.74	1.90	2.70	1.92	1.50	2.31	2.56	1.69	**	NS
**Maxillary skeletal**										
Co-point A (mm)	6.94	3.97	5.10	3.66	3.09	2.16	2.36	2.24	**	*
SNA (°)	0.41	2.11	0.49	1.35	− 0.75	2.07	− 0.06	1.04	NS	NS
Point A-Nasion perp (mm)	0.58	2.14	0.35	2.16	− 0.23	2.23	− 1.56	1.10	NS	**
Maxillary depth (°)	0.60	2.57	0.23	2.20	− 0.22	2.09	− 1.34	0.84	NS	*
**Mandibular skeletal**										
Co-Gn (mm)	11.92	5.13	10.58	6.77	6.38	4.06	7.64	3.77	**	NS
SNB (°)	0.00	2.28	0.06	1.12	0.11	1.60	0.74	1.59	NS	NS
Pg-Nasion perp (mm)	1.26	4.61	0.22	5.46	1.50	3.00	− 0.40	1.83	NS	NS
**Maxillary/mandibular**										
Wits appraisal (mm)	3.16	1.77	2.50	2.12	− 0.74	1.21	− 0.26	0.82	***	***
ANB (°)	0.72	1.58	0.48	1.84	− 0.85	1.18	− 0.91	1.12	**	*
Maxillary/mandibibular difference (mm)	5.02	3.50	5.28	4.81	3.62	2.20	4.37	2.16	NS	NS
**Vertical skeletal**										
FH-occlusal plane (°)	− 4.14	3.10	− 1.78	4.22	− 1.62	2.64	− 0.62	2.78	*	NS
SN-palatal plane (°)	− 0.46	2.19	− 0.80	2.05	1.49	3.15	− 0.96	1.84	NS	NS
MPA (°)	− 0.79	2.94	− 0.38	2.81	− 0.56	2.22	0.47	1.99	NS	NS
Nasion-Me (mm)	12.96	5.33	10.62	6.70	6.52	5.54	7.75	4.87	**	NS
S-Go (mm)	9.72	3.50	8.10	5.16	4.55	4.64	6.17	3.36	**	NS
ANS-Me (mm)	7.36	3.76	5.70	3.43	3.37	3.25	3.00	2.97	**	*
Gonial angle (°)	− 4.68	5.33	− 2.42	3.39	− 1.94	1.99	− 1.74	2.69	NS	NS
**Facial pattern**										
S-Go/Nasion-Me (%)	1.48	2.28	1.95	1.78	0.86	2.30	1.70	1.56	NS	NS
Facial axis (°)	− 1.33	2.86	− 0.88	2.27	− 0.46	1.56	− 0.26	1.53	NS	NS
**Interdental**										
Overjet (mm)	4.07	2.44	2.12	2.27	− 1.14	1.48	− 2.06	1.48	***	***
Overbite (mm)	1.20	1.55	0.05	2.40	− 0.85	1.89	− 1.14	1.50	**	NS
Interincisal angle (°)	− 10.61	14.34	− 5.38	6.06	− 6.90	11.73	− 8.72	13.10	NS	NS
Molar relationship (mm)	2.25	2.17	0.72	1.98	− 1.06	1.23	− 1.85	1.27	***	***
**Dentoalveolar**										
U1 to point A to Nasion perp (mm)	3.10	2.82	1.68	0.96	0.62	2.71	1.27	1.35	*	NS
U1 to FH (°)	16.79	28.64	4.82	6.59	3.66	10.42	3.86	7.09	NS	NS
L1 to A-Pg (mm)	0.01	1.53	0.52	1.50	1.65	2.07	2.33	1.55	*	**
L1 to MPA (°)	1.32	5.75	− 0.38	2.86	3.16	6.18	1.40	3.57	NS	NS
**Soft tissue relationship**										
Lower lip to E plane (mm)	− 0.86	2.00	− 0.93	1.89	− 0.83	2.06	− 0.92	1.42	NS	NS
Upper lip to E plane (mm)	− 0.17	2.28	− 0.97	2.47	− 1.44	2.12	− 1.92	1.37	NS	NS
Nasiolabial angle (°)	− 3.03	4.36	− 0.43	5.09	− 0.70	6.63	1.63	2.04	NS	NS

**Table 6:** Comparison of the differences by sex between Class III (EG, experimental group) and Class I (CG, control group) groups in period T1–T2. *Pg* Pogonion, *FH* Frankfurt Horizontal, *SN* Sella-Nasion, *MPA* mandibular plane angle, *Me* Menton, *S* Sella, *Go* Gonion, *ANS* anterior nasal spine, *U1* upper incisor, *L1* lower incisor, *E plane* Esthetic plane. * *p* < 0.05; ** *p* < 0.01; *** *p* < 0.001

Cephalometric measures	EG				CG				T1–T2 (***t*** test EG vs. CG)	
	Females		Males		Females		Males			
	Mean	SD	Mean	SD	Mean	SD	Mean	SD	Significance	Significance
**Cranial base**										
Cranial flexure (°)	0.36	2.14	− 0.02	1.55	0.56	1.38	0.68	1.46	NS	NS
Po localization (mm)	0.56	3.25	− 2.68	5.12	− 0.10	1.71	− 0.96	1.95	NS	NS
Anterior cranial base (mm)	2.08	2.74	2.71	2.69	1.60	1.41	3.87	2.64	NS	NS
Posterior cranial base (mm)	0.20	2.63	3.51	5.29	0.66	1.42	1.70	1.53	NS	NS
**Maxillary skeletal**										
Co-point A (mm)	3.26	3.67	5.84	1.081	3.17	2.16	4.51	2.08	NS	NS
SNA (°)	0.45	3.54	0.06	1.32	0.32	1.22	− 0.42	1.08	NS	NS
Point A-Nasion perp (mm)	0.86	1.83	0.58	2.97	1.26	1.55	− 0.38	1.19	NS	NS
Maxillary depth (°)	0.82	1.64	0.70	2.64	1.24	1.63	0.26	1.26	NS	NS
**Mandibular skeletal**										
Co-Gn (mm)	6.49	5.98	8.94	9.02	5.44	3.15	8.72	3.61	NS	NS
SNB (°)	0.41	2.59	1.65	2.32	0.16	1.47	− 0.53	1.71	NS	**
Pg-Nasion perp (mm)	2.65	3.49	3.84	4.96	3.37	4.28	1.16	2.30	NS	NS
**Maxillary/mandibular**										
Wits appraisal (mm)	− 0.46	1.94	− 2.70	2.53	0.16	0.80	0.73	1.25	NS	***
ANB (°)	− 0.18	1.27	− 1.37	1.06	0.23	0.88	0.20	1.16	NS	**
Maxillary/mandibibular difference (mm)	3.22	3.23	5.64	7.62	2.26	1.88	4.42	3.20	NS	NS
**Vertical skeletal**										
FH-occlusal plane (°)	− 1.22	2.46	− 1.09	3.55	− 2.38	2.64	− 1.22	2.26	NS	NS
SN-palatal plane (°)	0.45	2.12	− 0.55	3.05	− 0.80	2.67	2.29	2.94	NS	*
MPA (°)	− 1.47	2.95	− 1.96	2.88	− 1.93	2.81	− 1.82	2.18	NS	NS
Nasion-Me (mm)	4.24	6.33	10.24	19.48	3.56	2.52	8.46	3.08	NS	NS
S-Go (mm)	3.92	3.42	5.09	5.45	3.60	3.36	8.61	3.56	NS	*
ANS-Me (mm)	3.17	3.29	5.82	11.59	1.90	1.86	3.12	2.28	NS	NS
Gonial angle (°)	− 0.98	4.55	− 1.08	3.32	− 1.20	4.74	− 3.90	6.82	NS	NS
**Facial pattern**										
S-Go/Nasion-Me (%)	1.46	2.76	1.66	2.04	0.77	2.03	0.97	2.46	NS	NS
Facial axis (°)	0.88	4.30	1.87	2.68	1.06	1.49	0.79	1.20	NS	NS
**Interdental**										
Overjet (mm)	− 0.68	0.70	− 1.09	1.47	0.20	1.15	1.62	1.34	*	***
Overbite (mm)	− 0.38	0.75	− 0.64	0.90	0.74	1.31	0.84	1.02	**	***
Interincisal angle (°)	0.66	6.45	3.55	5.92	0.81	8.79	6.34	4.41	NS	NS
Molar relationship (mm)	− 0.54	1.23	− 0.76	1.76	− 0.02	0.81	− 0.12	1.11	NS	NS
**Dentoalveolar**										
U1 to point A to Nasion perp (mm)	0.88	1.62	1.14	0.87	1.14	0.85	1.12	1.19	NS	NS
U1 to FH (°)	− 0.48	3.98	− 0.06	2.85	0.80	5.76	0.02	2.43	NS	NS
L1 to A-Pg (mm)	0.52	1.39	0.32	1.21	0.12	1.47	− 0.72	1.52	NS	*
L1 to MPA (°)	1.27	3.72	− 1.13	4.20	1.15	4.41	− 0.51	1.81	NS	NS
**Soft tissue relationship**										
Lower lip to E plane (mm)	− 0.94	2.08	− 1.44	1.61	− 1.46	2.30	− 1.76	0.85	NS	NS
Upper lip to E plane (mm)	− 1.57	2.08	− 2.63	2.80	− 1.86	2.03	− 1.98	0.95	NS	NS
Nasiolabial angle (°)	− 0.16	5.68	− 1.06	4.36	0.76	4.74	− 0.43	2.10	NS	NS

In males, there is a larger increase in ACB (3.28 ± 2.16 mm), anterior facial height (ANS-Me) (5.70 ± 3.43 mm), and maxillary length (5.10 ± 3.66 mm), point A is protruded (0.35 ± 2.16 mm) while it is retruded in Class I (− 1.56 ± 1.10 mm), maxillary depth increases (0.23 ± 2.20°) while it decreases in Class I (− 1.34 ± 0.84°), the ANB angle and Wits appraisal increase (ANB: 0.48 ± 1.84°/Wits: 2.50 ± 2.12 mm) while they decrease in Class I (ANB: -0.91 ± 1.12°/Wits: − 0.26 ± 0.82 mm), overjet increases (2.12 ± 2.27 mm) while it decreases in Class I (− 2.06 ± 1.48 mm). Molar relationship improves from class III to class I in Class III males, while it tends towards class III in Class I males. The lower incisor is more protruded in Class I (2.33 ± 1.55 mm) than in Class III (0.52 ± 1.50 mm) subjects.

In the T1–T2 period (Table 6), for females, the only significant differences between the two groups were overjet and overbite, which were reduced in Class III (overjet: − 0.68 ± 0.70 mm/overbite: − 0.38 ± 0.75 mm) while they increased in Class I (overjet: 0.20 ± 1.15 mm/overbite: 0.74 ± 1.31 mm). Males show more significant differences: the SNB angle increases in Class III (1.65 ± 2.32°) while it decreases in Class I (− 0.53 ± 1.71°); Wits appraisal, the ANB angle, and the palatal plane angle are significantly reduced in Class III (Wits: − 2.70 ± 2.53 mm/ANB: − 1.37 ± 1.06/PP-SN: − 0.55 ± 3.05°); PFH increased less in Class III (5.09 ± 5.45 mm) than in Class I (8.61 ± 3.56 mm), overjet and overbite decreased in Class III (overjet:− 1.09 ± 1.47 mm/overbite: − 0.64 ± 0.90 mm) while they increased in Class I (overjet: 1.62 ± 1.34 mm/overbite: 0.84 ± 1.02 mm), and the lower incisor is more protruded in Class III (0.32 ± 1.21 mm) than in Class I (− 0.72 ± 1.52 mm).

## Discussion

The present study analysed the influence of sexual dimorphism on long-term stability (10 years) of skeletal Class III malocclusion treatment with RME combined with face mask protraction followed by fixed appliances. As far as we know, this is the first study that analyses differences between sex in the evolution of the treated Class III patients in such a long period of time (10 years post-treatment). In the Class III group, changes in the maxillomandibular relationship after treatment were favourable. Overjet and overbite relapse were observed in both females and males. In the long term, significant differences are observed between males and females, mainly in the ANB angle and Wits appraisal, which developed more negatively in males. The treatment success rate ten years later is 73.3% in males and 80% in females. Many cephalometric measurements have been analysed (*n*=34) and some of them are closely correlated. Therefore, it could be that some statistically significant differences may be found by chance, especially those with higher p values (*p < 0.05). Results for these comparisons should be taken with caution.

During treatment (T0–T1), very favourable skeletal changes were observed in the Class III group, both in males and females. This improvement in the intermaxillary relationship has been extensively described previously [11, 18, 21, 22, 24, 29–31]. These skeletal effects were due to significant changes in the maxilla and mandible. On the maxilla, advancement, increase in maxillary length, the advancement of point A, and increase in maxillary depth were observed, similar to what has been reported in other studies [22, 32, 33]. In contrast, in the Class I group, point A is retruded, more so in males. The effects on the mandible in the Class III group include retarded growth while the SNB angle remains stable, as reported in other studies [18, 21]; however, some authors even describe a decrease in the SNB angle during treatment [2, 22, 33]. The ANB angle and Wits appraisal increase during treatment, becoming more positive, both in males and females, unlike in the Class I group, in which the values of both variables decrease in both sexes. In the Class III group, the angle of the occlusal plane relative to the Frankfurt plane is reduced in females, with an anterior rotation of the maxilla; and also interdental relationships improve in both sexes, correcting overjet, overbite, and molar relationship. The upper incisor is buccally displaced during Class III treatment, to a greater extent in females, reflecting the dentoalveolar compensation of the upper incisor during treatment, while the lower incisor is less protruded in Class III than in Class I, both in females and males. Therefore, it is clear how buccal displacement of the lower incisor is prevented during treatment. These dental effects are similar to those described by other authors [18, 22, 29, 32], although some [7] describe a lingualization of the lower incisor during treatment that was not present in our study, as we only observed that the lower incisor remains in its lingualized position.

During treatment, the only variable differing between males and females is overjet. Overjet change from T0 to T1 is much more favourable in females, achieving an improvement of 4 mm on average during treatment, but only 2 mm in males. Therefore, it should be noted the better prognosis in females for Class III malocclusion treatment.

Analysis of the T1–T2 interval allowed us to observe long-term changes after Class III treatment. In the Class III group, substantial differences are observed between males and females, mainly in the ANB angle and Wits appraisal, which develop more negatively in males, indicating a worse prognosis in the stability of long-term outcomes and a greater tendency to relapse in males than in females. Furthermore, in males with Class III, the SNB angle increases, while it decreases in males with Class I. This increased SNB angle can be attributed to the remaining mandibular growth. The ANB angle and Wits appraisal become more negative, not reaching initial values (T0) but, as noted in the literature, mandibular growth distinctive of Class III continues, and skeletal and interdental relationship worsen again after the end of treatment [5, 18, 22, 24, 33].

Overjet and overbite relapse 10 years after Class III treatment, in both females and males. In females, they are the only two variables showing significant differences between Class III and Class I groups during this T1–T2 period. This post-treatment development in overjet and overbite is also observed in Class I but in the opposite direction. Overbite decreases in Class III and increases in Class I. In both class III and class I patients, overjet and overbite had a post-treatment development in a direction that was the opposite to their correction. Our study found that the interdental relationship relapse is higher in males than in females, which could be due to a larger overjet of the lower incisor in males during this period. Since, as mentioned in other studies [33], interdental relationship relapse can be expected, the possibility of applying overcorrection during treatment could be considered.

Our study found no maxillary relapse, like other authors, who also consider maxillary changes to be stable during treatment [7]; however, other studies have described a tendency to relapse in the anteroposterior position of the maxilla [22, 33].

The most common criteria for long-term class III relapse reported in different studies are class III molar relationship and anterior crossbite [1, 21, 22, 27, 28]. In our Class III patients, the success rate is 73.3% in males and 80% in females, similar to what has been described in previous studies. In the meta-analysis conducted by Lin et al. [33], the success rate in the studies reviewed ranges from 67 to 95%. Massucci et al. [22] observed 73%, Wells et al. [34] 75%, Westwood et al. [18] 76%, and Ngan et al. 75% [35]. In a previous study that only included females, we found a success rate of 81.8% [12]. A reason for the high percentage of stable cases in our sample could be due to the fact that the mandibular plane remained unchanged during treatment. According to various authors [21, 25], there is greater stability if the mandibular plane does not change during treatment.

It is important to tell the patients and their parents that prepuberal class III treatment is a very long treatment that requires overcorrection and a long retention period, especially in males. Long-term follow-up is crucial, even when the active growth period has finished.

## Limitations

Our study has some limitations, such as the study design (retrospective study) and the selection of a treated Class I control group. A sample of untreated Class III patients would have been ideal, but unfeasible for ethical reasons. Although most of the class III patients were at CS1, some of them were at a later stage at T0. Instead of “early treatment”, the term “prepuberal treatment” should be used instead. Finally, the sample size is small, which must be considered when interpreting the results. Further prospective studies are needed to examine the long-term stability of treatment outcomes and to compare the skeletal and dental effects on the craniofacial structure between males and females, especially in non-growing patients.

## Conclusions

There are significant differences in the long-term stability of Class III treatment outcomes between males and females, although some results should be taken with caution. Relapse of the intermaxillary relationship is higher in males, with larger worsening of the ANB angle and Wits appraisal. Treatment success rate after 10 years is higher in females (80%) than in males (73.3%).

Our findings related to sexual dimorphism in skeletal class III patients suggest a better prognosis in females than males both for treatment outcomes and the long-term post-treatment development. From a clinical point of view, more overcorrection during treatment as well as longer follow-up after treatment in males is suggested.

## Data Availability

The datasets generated and/or analysed during the current study are not publicly available due to our national Law on Data Protection, but are available from the corresponding author on reasonable request

## Abbreviations

A: Point A
ACB: Anterior cranial base
AFH: Anterior facial height
ANS: Anterior nasal spine
ANS-Me: Anterior facial height
Co: Condyleon
Co-A: Maxillary length (Condyleon-Point A)
Co-Gn: Mandibular length (Condyleon-Gnation)
CS: Cervical stage
FH: Frankfurt Horizontal
Gn: Gnation
Go: Gonion
IMPA: Incisor to mandibular plane angle
L1: Lower incisor
Me: Menton
MPA: Mandibular plane angle
OP: Occlusal plane
OP-FH: Occlusal plane angle
PCB: Posterior cranial base
perp: Perpendicular
PFH: Posterior facial height
PFH: Posterior facial height
Pg: Pogonion
Po: Porion
PP-SN: Palatal plane angle
RPE: Rapid maxillary expansion
S: Sella
SN: Sella-Nasion
U1: Upper incisor
